# The Dark Side of Expressed Humility for Non-humble Leaders: A Conservation of Resources Perspective

**DOI:** 10.3389/fpsyg.2019.01858

**Published:** 2019-08-22

**Authors:** Kejian Yang, Longzhi Zhou, Zhen Wang, Chen Lin, Zhengxue Luo

**Affiliations:** ^1^Fourth Military Medical University, Xi’an, China; ^2^School of Labor and Human Resources, Renmin University of China, Beijing, China

**Keywords:** expressed humility, honesty–humility, emotional exhaustion, turnover intentions, work-to-family conflict

## Abstract

Although existing studies to date predominately focus on the beneficial effects of leader expressed humility on followers, knowledge about how those behaviors impact the leaders themselves is scarce. Drawing on the conservation of resources theory, we develop and test a model that specifies for whom and how expressing humility has detrimental effects on leaders’ emotional exhaustion and the downstream implications of this effect for leaders’ turnover intentions and work-to-family conflict. Data from a multisource, time-lagged survey of 55 team leaders and 281 followers showed that expressed humility was positively associated with leaders’ emotional exhaustion when Honesty–Humility was low, after controlling for Emotionality, sleep quality, overall job satisfaction, and hindrance stressors. In addition, we found that expressed humility was positively and indirectly related to leaders’ turnover intentions and work-to-family conflict *via* emotional exhaustion when Honesty–Humility was low. Overall, our research sheds light on why and under what conditions the dark side of humble leader behaviors is going to emerge and take its toll on the leaders themselves. Theoretical and practical implications are discussed.

## Introduction

With the increasingly turbulent and unpredictable environment, organizations have to move beyond the “great man” or the hero myth perspectives of leaders who could figure it all out at the top (Marion and Uhl-Bien, 2001; Uhl-Bien et al., 2007). To attain long-term competitive advantages, organizations require leaders to have more humility, which means to show limitations and acknowledge others’ strengths and contributions (Morris et al., 2005). Accordingly, scholars have been showing great interest in the concept of leader expressed humility (Nielsen and Marrone, 2018; Wang et al., 2018). Expressed humility refers to an interpersonal characteristic that emerges in social contexts that is manifested by being willing to view oneself accurately; appreciating strengths and contributions of others; and being open to new advice, ideas, and feedback (teachability) (Owens and Hekman, 2012; Owens et al., 2013, 2015; Wang et al., 2018). Existing evidence has indeed shown that leader expressed humility manifested by admitting mistakes and limits, holding positive views of others, and having a desire to learn is associated with positive outcomes for followers (such as increased levels of engagement, positive affect, and performance; Owens et al., 2013; Wang et al., 2018); teams (such as higher team performance and innovation; Owens and Hekman, 2016; Rego et al., 2017; Hu et al., 2018); and organizations (such as increased levels of top management team integration, middle manager satisfaction, and organizational performance; Ou et al., 2017, 2018).

However, while prior studies have documented how leader expressed humility could exert benefits on followers’ affective and behavioral outcomes, it is unclear whether and in what ways engaging in humble behaviors impacts the leaders themselves, and further on, for whom and how expressed humility may take its toll on leaders. Failing to address the potential detriments of humble leader behavior is problematic because emerging research reveals that engaging in what are generally thought to be “good” leader behaviors also have depleting effects on leaders (Barling and Cloutier, 2017; Lin et al., 2018). Addressing this question is also important in that understanding the dark side of expressed humility helps depict a nuanced full-range picture of humble leader behaviors and accumulates knowledge about leader expressed humility that can be leveraged to aid leadership development.

To answer these questions, we draw on the conservation of resources (COR) theory and the counterdispositional framework from an actor-centric perspective to examine the possible detrimental impacts of engaging in humble leader behaviors for non-humble leaders. We argue that non-humble leaders (i.e., low on Honesty–Humility) could counterdispositionally express humility at work since they are dishonest and they have the purpose of influencing followers to reach desired goals (McCabe and Fleeson, 2012, 2016). However, acting counterdispositionally humble would cause resource loss (i.e., emotional exhaustion) and subsequent negative work-related outcomes (i.e., turnover intentions) and family-related outcomes (i.e., work-to-family conflict). Following previous research (Kammeyer-Mueller et al., 2016; Lin et al., 2018), we identify emotional exhaustion as a state of resource loss. The COR theory proposes that resource loss could result in psychological imbalance and, ultimately, lead to emotional exhaustion if not replenished. From the other side, resource acquisition is beneficial and could cut down emotional exhaustion (Hobfoll and Freedy, 1993). In addition, the COR theory suggests that when people experience resource loss at work, they tend to leave the current situation and quit their jobs to avoid further loss (Hobfoll, 2002; Swider and Zimmerman, 2010; Lin et al., 2018) and carry the frustrations of resource loss from work to the home domain and fail to fulfill family role demands (Carlson et al., 2012; Nohe et al., 2015). Past studies have shown that work-to-family conflict and turnover intentions could be affected by resource loss and represent primary outcomes of the COR theory (Halbesleben and Bowler, 2007; Carlson et al., 2012). Therefore, we entertain that leaders’ emotional exhaustion will in turn influence their turnover intentions and work-to-family conflict.

**FIGURE 1:**
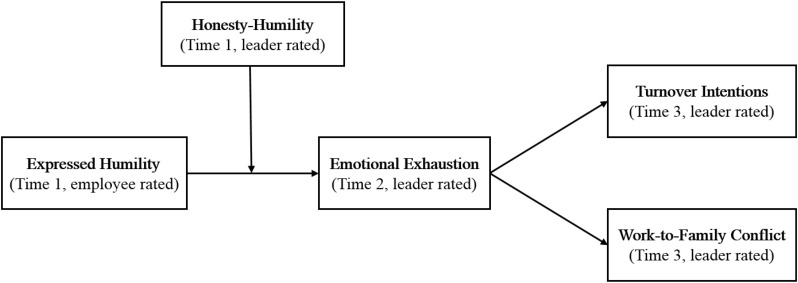
Theoretical model of the study.

By examining the hypothesized model in a time-lagged, multisource study, our paper makes several contributions. First, we shift the literature’s dominating focus on the effects of expressed humility from followers to leaders. While relevant literature constantly suggests a positive relationship between leader expressed humility and follower affective and behavioral outcomes (Owens et al., 2013; Wang et al., 2018), it remains unexplored whether this positive effect holds for the leaders themselves. Our study is an initial attempt to theorize and empirically investigate how and when expressing humility undermines leaders. In addition, our study contributes to the leader humility literature by investigating the boundary conditions under which humble leader behavior may impair leaders. Integrating the COR theory and literature on acting counterdispositionally, we argue that non-humble leaders ought to address the dissonance when acting counterdispositionally humble (Côté and Moskowitz, 1998; Brotheridge and Lee, 2002; Zelenski et al., 2012), which can be a resource-depleting process for them. Third, by echoing calls to understand the toll of enacting high-quality leadership on the well-being of the leaders themselves (Barling and Cloutier, 2017), we utilize the counterdispositional framework from an actor-centric perspective and examine for whom enacting humble leadership behaviors may result in their own emotional exhaustion, turnover intention, and work-to-family conflict. Over the past decades, there has been an increasing interest in the impact of high-quality leadership behaviors on followers’ work attitudes and well-being from a recipient-centric perspective (Inceoglu et al., 2018), whereby limited attention has been paid to the potential costs of such behaviors on leaders’ own work attitudes and well-being. This shortage is surprising given that leaders play a critical role in organizations and that their well-being is of great importance to themselves, their followers, and the organization (Quick et al., 2007). Finally, our research also contributes to the COR theory by identifying humble leadership behavior, a so-called “good” behavior, as an important event involving the detrimental process of resource loss when it is enacted out of character. Past research has focused exclusively on investigating how so-called good behaviors, such as voice and helping behaviors, facilitate resource conservation and generation (Qin et al., 2014; Koopman et al., 2016). Our research highlights the importance of taking individual factors in general and personality in particular into consideration in the research of when good behaviors generate/deplete resources. A depiction of our hypothesized model is shown in Figure 1.

## Theory and Hypotheses

### Emotional Exhaustion by Acting Counterdispositionally Humble

Before elaborating on the consequences, we must consider whether and why leaders could act counterdispositionally humble. Research based on functional perspectives has revealed that leaders could vary their behaviors across situations (Santos et al., 2015; Liu et al., 2017a), counterdispositionally enact specific trait manifestations (Wang and Campbell, 2018), or behave in a paradoxical manner (Rosing et al., 2011; Zhang et al., 2015b). For example, a self-centered, narcissistic leader could act others-centered and humble to motivate followers and boost performance and innovation (Owens et al., 2015; Zhang et al., 2017). One major difference of the HEXACO model of personality from the Big Five or Five-Factor Model is its inclusion of an Honesty–Humility dimension (Breevaart and de Vries, 2017; Ashton and Lee, 2018). Honesty–Humility is generally defined as “the tendency to be fair and genuine in dealing with others, in the sense of cooperating even when one might exploit them” (Ashton and Lee, 2007). People high on Honesty–Humility have a tendency to be genuine and humble in interpersonal relations (Lee and Ashton, 2004; Ashton and Lee, 2008; Oh et al., 2011). They tend to view themselves as ordinary people without any claim of entitlement and privileges (Lee and Ashton, 2004; de Vries, 2013). In contrast, people low on Honesty–Humility tend to manipulate and cheat others in the interest of personal gain (Ashton et al., 2014; Lee and Ashton, 2018). Considering this, it is reasonable to imagine that dishonest leaders who have a low level of Honesty–Humility are prone to act and regulate their behaviors to express humility (de Vries, 2018). In addition, non-humble leaders may express humility because of role demands posed by organizations. Organizations are increasingly embracing humble leadership due to the ambiguity and complexity of the environment (Morris et al., 2005; Uhl-Bien et al., 2007; Owens and Hekman, 2012). Leaders need to take advice from followers, acknowledge others’ strengths, and ultimately mobilize the whole organization. In addition, leaders low on Honesty–Humility may act humbly for the purpose of desired goals. Research has revealed that people could act counterdispositionally when pursuing personal goals (McCabe and Fleeson, 2012, 2016). In addition, expressed humility has been shown to be positively related to team performance and innovation (Owens and Hekman, 2016; Rego et al., 2017; Hu et al., 2018). Leaders low on Honesty–Humility may identify expressing humility as a tool in the pursuit of better leadership appraisal since team performance and innovation can be indicators of leader effectiveness. More directly, a small insignificant correlation (*r* = 0.20, *p* > 0.05) between self-reported humility and expressed humility has been found in an empirical study (Rego et al., 2017), supporting the idea that non-humble leaders could act counterdispositionally humble. Thus, we suggest that it is possible that leaders low on Honesty–Humility could be perceived as having a high level of expressed humility by their followers.

Honesty–Humility represents individual differences in manipulation and deception (Ashton and Lee, 2008), so it might be intuitive for dishonest leaders (low on Honesty–Humility) to behave in deceptive ways to manage their impressions and behave as effective leaders. However, when such effective leadership behaviors have to be humble behaviors, leaders low on Honesty–Humility might be resource depleted since enacting humble behaviors is out of their character (Zelenski et al., 2012, 2013). The core premise of the COR theory is that people make every effort to obtain, protect, and retain the resources that are valued by the individuals (Hobfoll, 1989; Hobfoll and Shirom, 2001; Halbesleben et al., 2014; Hobfoll et al., 2018). From the COR perspective, we thus propose that acting counterdispositionally humble necessitates the expenditure of resources, which could result in emotional exhaustion that reflects the primary results of resource loss (Halbesleben et al., 2014).

First, behaving in ways that contradict dispositions might deplete leaders’ self-control resources (Zelenski et al., 2012, 2013). Regardless of leaders’ specific personal goals, there are presumably stable internal factors that contribute to Honesty–Humility. Leaders who act counter to these internal tendencies ought to exert effortful control of their behaviors to override the automatic tendencies. Second, acting out of character requires emotional labor, especially surface acting. Acting counterdispositionally entails surface acting, which means that the actor has to portray emotions that are not actually felt (Grandey, 2000). The behavioral concordance model has suggested that people would experience a negative affect when engaging in behaviors that are opposite to their traits (Moskowitz and Coté, 1995; Côté and Moskowitz, 1998; Pickett et al., 2019). As such, dishonest leaders ought to engage in emotional labor to suppress the negative affect originated from acting counterdispositionally humble. Displaying proper emotions that misalign with their true feelings would deplete their personal resources (Brotheridge and Lee, 2002; Bono and Vey, 2007). Despite the fact that deep acting has proved to be better than surfacing acting for well-being, both types of emotional labor necessitate the expenditure of effort and energy, leading to increased emotional exhaustion (Hülsheger and Schewe, 2011). Finally, although leaders low on Honesty–Humility are prone to behave in a deceptive way, counterdispositionally expressing humility might contradict their true self. Based on this counterdispositional explanation, acting counterdispositionally humble might impair the sense of authenticity among leaders low on Honesty–Humility, which has a depleting effect on their psychological resources (Gardner et al., 2009). Acting inconsistent with the inner self entails self-regulation effort, which continuously drains an individual’s mental resources (Brotheridge and Lee, 2002; Gardner et al., 2009). Brotheridge and Lee (2002) have found that authenticity was negatively related to emotional exhaustion. Similarly, Weiss et al. (2018) found that authentic leader behaviors that were consistent with their inner feelings and thoughts were positively related to leaders’ mental well-being.

Beyond the counterdisposition explanation mentioned above, it may also be true that deceptively expressing humility could directly lead to emotional exhaustion. Despite it being behaviorally concordant for leaders low on Honesty–Humility to behave in a deceptive manner (Ashton and Lee, 2008), faking behaviors consumes much time and energy of their own. Specifically, humble leader behaviors may be particularly resource consuming given the range and number of complex tasks and behaviors required for enacting humble leader behaviors. For instance, admitting limitations to model a growth orientation for followers is likely to expend time and energy (Owens and Hekman, 2012), and expressing positive emotions to acknowledge others’ strengths and show openness to feedback may require emotional regulation and increase emotional exhaustion (Hülsheger and Schewe, 2011). As such, this “deceptive behavior” explanation along with the counterdisposition explanation implies that being a leader low on Honesty–Humility who is perceived as humble by one’s subordinates could lead to emotional exhaustion of the leader himself/herself. Taking these together, we predict the following:

*Hypothesis 1*: Honesty–Humility will moderate the relationship between expressed humility and emotional exhaustion, such that there will be a positive relationship between expressed humility and emotional exhaustion when Honesty–Humility is low.

### Implications for Turnover Intentions and Work-to-Family Conflict

Beyond the direct implications of leaders’ expressed humility and Honesty–Humility for their emotional exhaustion, it is important to consider the downstream implications of resource loss for work- and family-related outcomes. Here, we focus on turnover intentions and work-to-family conflict because they are resource relevant (Nohe et al., 2015; Lin et al., 2018), which accords with the COR perspective.

The COR theory posits that resource loss is more harmful than resource gain (Halbesleben et al., 2014; Hobfoll et al., 2018). When people are exposed to resource loss, they strive to conserve their resources by adopting avoidance and withdrawal coping strategies to avoid further loss (Hobfoll and Shirom, 2001; Hobfoll et al., 2018). To protect resources and prevent them from further loss, people tend to engage in avoidant and withdrawal behaviors, such as fleeing from their current situations and quitting their jobs (Swider and Zimmerman, 2010). Indeed, there is ample evidence suggesting that emotional exhaustion is positively related to turnover intentions (Wright and Cropanzano, 1998; Houkes et al., 2003; Lin et al., 2018). Taking these together, acting counterdispositionally humble may leave non-humble leaders emotionally exhausted, and leader emotional exhaustion would subsequently generate intentions of quitting. Accordingly, we predict the following:

*Hypothesis 2a*: The indirect effects of expressed humility on turnover intentions *via* emotional exhaustion will be moderated by Honesty–Humility, such that expressed humility will have positive indirect effects on turnover intentions when Honesty–Humility is low.

Work-to-family conflict occurs when the demands of the work role deplete personal resources, thereby leaving inadequate resources to fulfill the family role (Voydanoff, 2004a, b; Byron, 2005). According to the COR theory, people have a limited reservoir of personal resources (e.g., time, energy, emotions) with which to deal with their surroundings (Hobfoll, 1989; Hobfoll and Shirom, 2001). When people experience resource loss, they have insufficient resources to manage work and family demands (Carlson et al., 2012). These depleted individuals have little energy for family chores or enrichment, thereby increasing work-to-family conflict. In fact, studies have confirmed that emotional exhaustion contributes to greater work-to-family conflict (Michel et al., 2011; Carlson et al., 2012). Overall, then, resource loss caused by acting counterdispositionally may spill over from work to home domains, and leader emotional exhaustion would subsequently lead to increased work-to-family conflict. Accordingly, we predict the following:

*Hypothesis 2b*: The indirect effects of expressed humility on work-to-family conflict *via* emotional exhaustion will be moderated by Honesty–Humility, such that expressed humility will have positive indirect effects on work-to-family conflict when Honesty–Humility is low.

## Materials and Methods

### Participants and Procedure

To examine our hypotheses, we collected data from leaders and followers in an electronic product design and manufacturing company located in western China. With the support from the organization’s top managerial officers and the assistance of the human resource department, we distributed pencil–paper surveys to the organization’s team leaders as well as their followers. The author team articulated the purpose and potential contributions of this research but did not disclose any specific hypotheses to them, and assured them that their responses would be kept confidential and only be used for research.

The data collection was organized into three phases, conducted at least 2 weeks apart. A researcher-assigned identification number was used for matching data from separate phases. During phase one, team leaders reported their Honesty–Humility, overall job satisfaction, sleep quality, and hindrance stressors. Followers, in turn, rated their leaders’ humble behaviors. At phase 2, leaders rated their emotional exhaustion, and during phase 3, leaders reported their turnover intentions and work-to-family conflict. In phase one, surveys were handed out to all leaders and followers within the company, for a total of 91 leader surveys and 486 follower surveys. Of these, 68 leader surveys (74.73%) and 356 follower surveys (73.25%) were returned. Finally, a total of 55 leaders completed all three phases, for a final leader response rate of 60.44% and a total of 281 matched leader–follower dyads. The mean team size was 5.11.

In the final sample, 49.10% of the leaders were male and an average of 43.31 years old. Leaders also averaged 21.39 years of tenure at their company. In total, 38.8% of subordinates were female. Subordinates were 37.13 years old on average, with an average of 16.28 years with the organization.

### Measures

All survey items were translated into Chinese following the standard translation and back-translation procedures (Brislin, 1986).

#### Expressed Humility

Humble leader behaviors were measured by aggregating subordinates’ ratings of their leaders *via* Owens et al. (2013) nine-item scale. Sample items include “My leader takes notice of others’ strengths,” “My leader actively seeks feedback, even if it is critical,” and “My leader is open to the advice of others” (1 = strongly disagree, 7 = strongly agree). Within-Group Interrater Reliability (*R*_wg_), Intra-Class Correlation 1 (ICC1), and Intra-Class Correlation 2 (ICC2) were examined to justify the appropriateness of aggregating leader expressed humility to the team level (Bliese, 2000). The results demonstrated that the mean *r*_wg_ value was 0.95, ICC1 = 0.23, ICC2 = 0.60, and the *F* value for analysis of variance (ANOVA) was highly significant in terms of between-team variances (*F*[54, 226] = 2.49, *p* < 0.01), which provided support for aggregating this construct to the team level.

#### Honesty–Humility

Leader Honesty–Humility was measured with the 10-item measure of Honesty–Humility included in the HEXACO-60, a brief personality inventory that measures the HEXACO model of personality structure (Ashton and Lee, 2009). Sample items include “Having a lot of money is not especially important to me” and “I think I am entitled to more respect than the average person is” (reverse-coded) (1 = strongly disagree, 5 = strongly agree).

#### Emotional Exhaustion

Emotional exhaustion was measured with the five-item subscale from the Maslach Burnout Inventory (Maslach et al., 1996). Leaders were asked to consider the past week when filling out this scale. Sample terms include “I feel burned out at my work” and “Working all day is really a strain for me” (1 = never, 7 = daily).

#### Turnover Intentions

Turnover intentions were measured *via* three items from Konovsky and Cropanzano (1991) scale. Sample terms include “How likely is it that you will look for a job outside of this organization during the next year?” and “If it were possible, how much would you like to get a new job?” (1 = very unlikely, 7 = very likely).

#### Work-to-Family Conflict

Work-to-family conflict was measured *via* five items from Netemeyer et al. (1996) scale. Sample terms include “The demand of my work interferes with my home and family life” and “My job produces strain that makes it difficult to fulfill family duties” (1 = strongly disagree, 7 = strongly agree).

#### Control Variables

Since our research is non-experimental, extraneous (i.e., third) variables may increase the concerns of contaminating the assessment and producing confounded relationships among study variables. In addition, controlling for third variables could help build the incremental validity between a predictor and a criterion (Bernerth et al., 2018). Therefore, to account for any demographic differences in emotional exhaustion, turnover intentions, and work-to-family conflict (Frone, 2000; Houkes et al., 2003; Bhave et al., 2010), we controlled for leaders’ ages, genders, and organizational tenures. We also controlled for Emotionality (Zimmerman, 2008; Blanch and Aluja, 2009; Kiffin-Petersen et al., 2011), sleep quality (Williams et al., 2006; Söderström et al., 2012), overall job satisfaction (Porter et al., 1974; Kinnunen et al., 2004), and hindrance stressors at work (Podsakoff et al., 2007; Culbertson et al., 2009) for their potential effects on emotional exhaustion, turnover intentions, and work-to-family conflict to build the incremental validity of the leader expressed humility and Honesty–Humility interaction and to confirm that the expressed humility and Honesty–Humility interaction is indeed attributable to emotional exhaustion, turnover intentions, and work-to-family conflict rather than other related constructs (Friedrich et al., 2009; Bernerth et al., 2018).

Emotionality was measured with the 10-item measure of Emotionality included in the HEXACO-60 (Ashton and Lee, 2009). Sample items include “I feel like crying when I see other people crying” and “I sometimes can’t help worrying about little things” (1 = strongly disagree, 5 = strongly agree). Sleep quality was measured *via* a single item validated by Liu et al. (2017b). Leaders were asked to rate how well they slept during the past few weeks using a seven-point Likert scale. Overall job satisfaction was measured by a three-item measure developed by Cammann et al. (1979). Sample terms include “In general, I don’t like my job” (reverse-coded) (1 = strongly disagree, 7 = strongly agree). Leaders were also instructed to report the extent to which they experience hindrance-related stress at work *via* Cavanaugh et al. (2000) five-item scale. Sample terms include “degree to which politics rather than performance affects organizational decisions” (1 = produces no stress, 7 = produces a great deal of stress).

## Results

Table 1 presents the descriptive statistics, alpha reliabilities, and correlations of the study variables. All scale reliabilities exceeded the criterion of 0.70 suggested by Nunnally (1978).

**TABLE 1:** Descriptive Statistics, Reliabilities, and Correlations of Variables.

		**Mean**	**SD**	**1**	**2**	**3**	**4**	**5**	**6**	**7**	**8**	**9**	**10**	**11**	**12**
1	Age	43.31	6.86												
2	Gender^a^	0.49	0.51	–0.06											
3	Tenure	21.39	12.21	0.60^∗∗^	−0.29^∗^										
4	Sleep quality	5.31	1.39	0.06	–0.01	–0.14									
5	Overall job satisfaction	5.28	1.10	–0.09	0.21	–0.18	0.18	**(0.85)**							
6	Hindrance stressors	2.84	0.94	0.27^∗^	−0.28^∗^	0.24	–0.15	–0.47^∗∗^	**(0.78)**						
7	Emotionality	2.37	0.61	0.02	−0.34^∗^	0.17	–0.22	−0.32^∗^	0.51^∗∗^	**(0.78)**					
8	Aggregated leader humility	5.02	0.58	−0.33^∗^	–0.11	–0.04	0.02	0.09	0.10	0.33	**(0.90)**				
9	Honesty–Humility	3.43	1.13	0.07	–0.15	0.12	–0.06	−0.34^∗^	0.10	–0.05	–0.15	**(0.91)**			
10	Emotional exhaustion	3.51	1.19	–0.21	–0.14	–0.04	–0.13	–0.06	0.07	0.24	0.23	–0.21	**(0.91)**		
11	Work-to-family conflict	2.97	1.10	–0.12	–0.14	–0.09	0.03	–0.20	0.35^∗∗^	0.41^∗∗^	0.21	–0.07	0.64^∗∗^	**(0.91)**	
12	Turnover intentions	3.04	1.52	–0.08	–0.20	–0.04	0.01	−0.28^∗^	0.26	0.40^∗∗^	0.30^∗^	–0.09	0.45^∗∗^	0.60^∗∗^	**(0.81)**

**N* = 55. ^*a*^Gender (0 = female, 1 = male). Scale reliabilities are in parentheses and bold. ^∗^*p* < 0.05, ^∗∗^*p* < 0.01 (two-tailed).*

Due to the hierarchical data structure of our research (multiple subordinates belong to one leader), it is necessary to account for multilevel variability when conducting factor analysis (Dyer et al., 2005). Therefore, multilevel confirmatory factor analyses (CFAs) were conducted to evaluate the discriminant validity of study variables. Specifically, we set leader expressed humility at both level 1 (follower level) and level 2 (team/leader level) and set other variables at level 2 (including Honesty–Humility, emotional exhaustion, work-to-family conflict, turnover intentions, Emotionality, job satisfaction, and hindrance stressors). Due to our limited sample size at level 2, which makes our hypothesized model exceed the recommended ratio of parameter to sample size at level 2 (Bentler and Chou, 1987), the item parceling method was adopted to simplify the multilevel CFA models (Liu et al., 2017b). We created three parcels for expressed humility by combining items assessing three kinds of humble behaviors (i.e., willingness to view oneself accurately, appreciating others’ strengths and contributions, and teachability; Owens et al., 2013), and four parcels for Honesty–Humility and Emotionality by combining items assessing four different facets of the Honesty–Humility domain (i.e., sincerity, fairness, greed avoidance, and modesty; Ashton and Lee, 2009) and Emotionality domain (i.e., fearfulness, anxiety, dependence, and sentimentality; Ashton and Lee, 2009). In addition, we created three balanced parcels for emotional exhaustion, work-to-family conflict, and hindrance stressors, adopting the single-factor method by pairing off items with the highest and lowest factor loadings assigned to the first parcel, the second-highest and lowest factor loadings assigned to the second parcel, and continuing pairing until items were exhausted (Landis et al., 2000). In addition, we did not create parcels for turnover intentions and job satisfaction, because they only include three items. The results demonstrated that the hypothesized eight-factor model showed an acceptable fit to data [χ*2* (281) = 499.61, *p* < 0.01, χ*2/df* = 1.78, Tucker-Lewis index (*TLI*) = 0.90, Comparative fit index (*CFI*) = 0.90, Root mean square error of approximation (*RMSEA*) = 0.05, Standardized root mean square residual (SRMR) (between) = 0.07], and all loadings were significant (*p* < 0.05). This model fits the data significantly better than other alternative models, including a seven-factor model in which work-to-family conflict and turnover intentions were set to load on a single factor (Δχ*2*_(__6__) =_ 41.45, *p* < 0.01), a six-factor model in which Honesty–Humility, job satisfaction, and hindrance stressors were set to load on a single factor (Δχ*2*_(__11__) =_ 156.72, *p* < 0.01), and a three-factor model in which Honesty–Humility, emotional exhaustion, turnover intentions, work-to-family conflict, job satisfaction, and hindrance stressors were set to load on a single factor (Δχ*2*_(__20__) =_ 428.58, *p* < 0.01). Those results demonstrated the discriminant validity of our measures.

**FIGURE 2:**
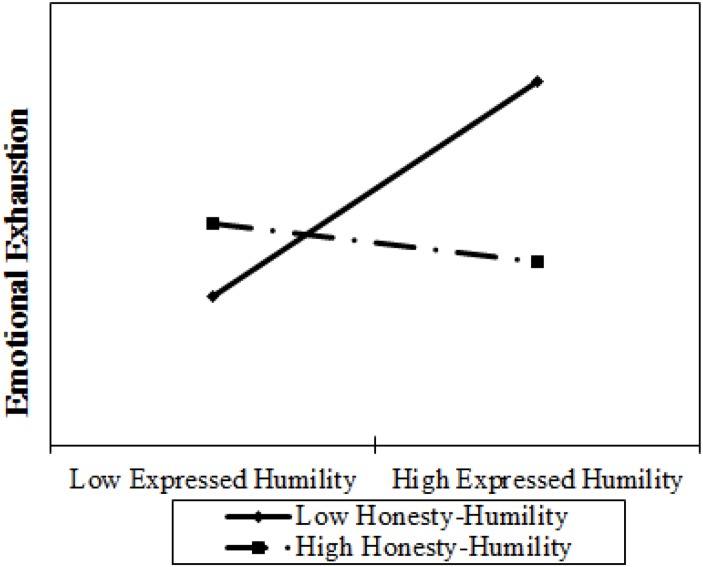
The interactive effect of expressed humility and Honesty–Humility on emotional exhaustion.

Before testing the specific hypotheses, we standardized leader expressed humility and Honesty–Humility and created the interaction term (expressed humility X Honesty–Humility) using the standardized variables. First, we tested Hypothesis 1 by examining the interactive effect of expressed humility and Honesty–Humility on emotional exhaustion. As presented in Model 2 of Table 2, results of multivariate regressions provided support for the anticipated effect, with the interaction term explaining an additional 9% of variance in emotional exhaustion (β = −0.36, *p* = 0.03) after controlling for all control variables. And results remain similar when omitting all control variables (Model 3 in Table 2). The interaction is plotted in Figure 2. Simple slopes tests indicated that leader expressed humility was positively related to emotional exhaustion when Honesty–Humility was low (β = 0.83, *SE* = 0.36, *p* = 0.02), unrelated to emotional exhaustion when Honesty–Humility was at the mean level (β = 0.34, *SE* = 0.21, *p* = 0.11) or high (β = −0.16, *SE* = 0.22, *p* = 0.48). Thus, Hypothesis 1 was supported.

**TABLE 2:** Estimated Coefficients of the Moderated Mediation Model.

**Variables**	**Emotional exhaustion**			**Turnover intentions**			**Work-to-family conflict**		
			
	**M1**	**M2**	**M3**	**M4**	**M5**	**M6**	**M7**	**M8**	**M9**
**Control variables**									
Age	–0.23	–0.18		–0.05	0.14		–0.13	0.00	
Gender	–0.08	–0.09		–0.07	–0.06		0.01	0.07	
Tenure	0.04	0.04		–0.12	–0.18		–0.11	–0.14	
Emotionality	0.20	0.03		0.35^∗^	0.22		0.33^∗^	0.26^†^	
Sleep quality	–0.07	–0.15		0.11	0.12		0.14	0.22^†^	
Overall job satisfaction	0.02	–0.21		–0.18	–0.22		–0.03	0.03	
Hindrance stressors	–0.01	–0.08		0.04	0.03		0.25	0.28^∗^	
**Main predictors**									
Expressed humility		0.28	0.32^∗^		0.14	0.18		–0.09	0.07
Honesty–Humility		–0.18	–0.11		–0.07	0.02		0.07	0.08
Expressed humility X Honesty–Humility		−0.36^∗^	−0.29^∗^		0.10	0.07		0.12	0.01
Emotional exhaustion					0.39^∗∗^	0.42^∗∗^		0.66^∗∗^	0.64^∗∗^
**F**	0.85	1.52	3.13	1.92	2.49	4.06	2.36	5.77	9.14
***R*^2^**	0.11	0.27	0.16	0.22	0.39	0.25	0.26	0.60	0.42
**Δ*R*^2^**		0.14^∗^			0.17^∗∗^			0.34^∗∗^	

**N* = 55. All coefficients are standardized. ^†^*p* < 0.10, ^∗^*p* < 0.05, ^∗∗^g*p* < 0.01 (two-tailed).*

Hypothesis 2a and Hypothesis 2b proposed a conditional indirect effect of expressed humility on work-to-family conflict and turnover intentions *via* emotional exhaustion, moderated by Honesty–Humility. We followed the methods suggested by Hayes (2013) to examine conditional indirect effects. This method is based on tests of products of coefficients and does not assume the products to be normally distributed, thus having more statistical power than traditional methods. We applied the bias-corrected bootstrapping procedure and bootstrapped 20,000 estimations of the indirect effects of expressed humility X Honesty–Humility on work-to-family conflict and turnover intentions *via* emotional exhaustion. In addition, we reported the index of moderated mediation, which directly provides an inferential examination of the conditional indirect effect (Hayes, 2015). The index of moderated mediation functions as a direct estimator to linearly associate the indirect effect with the values of a moderator and to imply if any specific conditional indirect effects determined by the distinct values of the moderator are significantly different.

As presented in Model 5 of Table 2, the results of the multivariate regressions showed that emotional exhaustion was positively associated with turnover intentions (β = 0.39, *p* < 0.01) after controlling for all first-stage effects and control variables. In addition, the results remain similar when omitting all control variables (Model 6 in Table 2). The indirect effect of leader expressed humility on work-to-family conflict *via* emotional exhaustion was significant and positive when Honesty–Humility was low (*indirect effect* = 0.39, *SE* = 0.26, Confidence Interval (CI) [0.02, 1.07]) and not significant when leader Honesty–Humility was at the mean level (β = 0.16, *SE* = 0.12, CI [−0.01, 0.50]) or high (β = −0.07, *SE* = 0.10, CI [−0.38, 0.05]). The index of moderated mediation was significant as well, again demonstrating a meaningful role of Honesty–Humility in the effects of leader expressed humility on work-to-family conflict *via* emotional exhaustion (*index of moderated mediation* = −0.23, *SE* = 0.15, CI [−0.64, −0.02]). Thus, Hypothesis 2a was supported.

As presented in Model 8 of Table 2, results of multivariate regressions showed that emotional exhaustion was positively associated with work-to-family conflict (β = 0.66, *p* < 0.01) after controlling for all first-stage effects and control variables. In addition, the results remain similar when omitting all control variables (Model 9 in Table 2). The indirect effect of leader expressed humility on turnover intentions *via* emotional exhaustion was significant and positive when Honesty–Humility was low (*indirect effect* = 0.46, *SE* = 0.22, CI [0.07, 0.95]) and not significant when leader Honesty–Humility was at the mean level (β = 0.18, *SE* = 0.12, CI [−0.02, 0.45]) or high (β = −0.09, *SE* = 0.11, CI [−0.34, 0.09]). The index of moderated mediation was significant as well, again demonstrating a meaningful role of Honesty–Humility in the effects of leader expressed humility on turnover intentions *via* emotional exhaustion (*index of moderated mediation* = −0.27, *SE* = 0.13, CI [−0.57, −0.05]). Thus, Hypothesis 2b was supported.

## Discussion

Based on the COR theory, we developed and examined a model explaining for whom and how expressing humility affects leaders’ resource loss and work- and family-related consequences. Findings from a time-lagged, multisource field study revealed that emotional exhaustion mediated the interactive effect of leaders’ expressed humility and Honesty–Humility on their turnover intentions and work-to-family conflict, such that expressed humility was associated with an increase in emotional exhaustion when combined with low Honesty–Humility, which in turn leads to increased turnover intentions and work-to-family conflict.

However, we found that when leaders high on Honesty–Humility behave less humbly than normal, they do not experience an increase in emotional exhaustion. This asymmetrical finding is consistent with previous research in that they all found that not all counterdispositional behaviors have a detrimental effect on the actors (Zelenski et al., 2012; Pickett et al., 2019). One possible reason for the asymmetrical finding in this study might be that expressing humble behaviors is in itself an effortful task for leaders themselves (Owens and Hekman, 2012). It’s easy for leaders to behave in a powerful and authoritative manner (Zhang et al., 2015a); however, they have to exert constant effort to enact humble leader behaviors. Relative to counterdispositionally expressing humility, behaving less humbly than normal would be more natural for leaders, and this has little effect on their level of emotional exhaustion.

## Theoretical and Practical Implications

Our study has several key theoretical and practical contributions. First, we contribute to the leader expressed humility literature by broadening scholars’ understanding of the consequences of expressed humility. While previous studies have confirmed the beneficial effect of leader expressed humility on followers (Owens et al., 2013; Wang et al., 2018), the impacts of expressed humility for the leaders themselves have been largely overlooked. We developed a theoretical model suggesting that leader expressed humility may bring costs to non-humble leaders through the lens of the COR theory (Hobfoll, 1989; Hobfoll and Shirom, 2001; Halbesleben et al., 2014; Hobfoll et al., 2018). In support of our theory, we found that the interaction of leader expressed humility and Honesty–Humility had a positive indirect effect on leaders’ turnover intentions and work-to-family conflict through emotional exhaustion.

Second, we contribute to the COR theory by identifying that acting counterdispositionally is an important process involving resource loss. While previous studies have established that behaving in ways that contradict dispositions was related to a negative affect and ego depletion (Moskowitz and Coté, 1995; Côté and Moskowitz, 1998; Zelenski et al., 2012), the resource-related consequences of acting counterdispositionally have escaped nuanced examinations. Drawing on the COR theory, we argue that acting counterdispositionally entails an expenditure of resources and produces resource loss. The results demonstrated that when leaders low on Honesty–Humility expressed humility, they experienced resource loss, adopted avoidance coping strategies, and failed to fulfill family role demands.

From a practical standpoint, our finding that behaving humbly can lead to resource depletion when leaders are actually non-humble is noteworthy. With previous studies consistently revealing that leader expressed humility is associated with team performance and team innovation (Rego et al., 2017; Hu et al., 2018) and that humility can be seen as a virtue or behavioral pattern that can be taught (Morris et al., 2005; Owens et al., 2013), organizations increasingly require leaders to show shortcomings, acknowledge contributions of followers, and solicit advice from followers. However, we suggest caution in recommending that organizations provide training to every leader on how to express humility. In addition, research has found that intrinsic motivation could mitigate the negative effects of depletion (Babakus et al., 2008; Rubino et al., 2009); thus, a possible tactic for reducing the depleting effects of acting counterdispositionally humble is to improve non-humble leaders’ intrinsic motivations.

## Limitations and Future Research

Although our study adopted a time-lagged and multisource study design, it still has several limitations worth noting. First, although our research design reduced concerns of common method bias, the nature of our study precludes us drawing any causal inferences. There might exist other endogenous variables, beyond our control, explaining the relationships of our focal measurements. Thus, experimental research designs are needed. Alternatively, more robust statistical frameworks, particularly the instrumental variable estimations, would help address the endogeneity concerns (Bollmann et al., 2019). Second, we provided two parallel explanations (the counterdisposition explanation and the deceptive behavior explanation) regarding why being perceived as humble by ones’ subordinates while being a leader low on Honesty–Humility might be linked to emotional exhaustion. Despite them both implying that it would be emotionally exhausting for leaders low on Honesty–Humility to behave in a humble manner, we did not clarify which explanation is more important and likely. Future research would benefit by adopting a sounder theoretical framework or more nuanced research design to disentangle this issue. Third, we adopted the counterdisposition framework in explaining why acting counterdispositionally humble is related to emotional exhaustion; however, all existing evidence linking counterdispositional behaviors and well-being are irrelevant to Honesty–Humility. Given that we cannot guarantee that a phenomenon observed with one’s personality traits could fully generalize to any other trait, future research may have to provide more empirical evidence specific to Honesty–Humility to make the counterdisposition explanation more plausible. Fourth, although we collected data from different sources (leaders and followers), the final sample size of leaders was quite small (*N* = 55). It is well established that an inadequate sample size may lead to biased parameters (Schönbrodt and Perugini, 2013). We encourage future studies to replicate our study using a larger sample size. Fifth, all participants of this research come from a state-owned organization in China. As such, some might question the rationality of turnover intentions as a major outcome in our study, since there is low mobility of personnel in China’s state-owned enterprises, which is also indicated by the long tenure in our research. On one side, it would be more frustrating for leaders when they cannot quit their current job while having thoughts of quitting. Although they cannot quit their jobs, they can withdraw from their leadership responsibilities, displace their frustrations, and even supervise abusively. On the other side, this might suggest that our findings are more conservative because of the long tenure and relatively low value of leaders’ turnover intentions. All in all, these facts limit the generalizability of our findings to private enterprises in China and to organizations of other cultures. Sixth, we focused our examination on expressed humility and did not contemplate other leadership behaviors that might also bring detriments to leaders low on Honesty–Humility themselves. Hereafter, it is a valid research question whether our logic would hold for other “good” leadership behaviors such as transformational leadership, ethical leadership, and servant leadership. Finally, another logical extension of our study is to examine whether acting counterdispositionally humble produces resource loss, which in turn negatively predicts future humble leader behaviors. Future research could benefit by adopting a cross-lagged or longitudinal study design to see what the motives and barriers of leader expressed humility are.

## Conclusion

Although leader expressed humility brings benefits to followers, teams, and firms, our study demonstrates for whom and how it may be detrimental to the leaders themselves. Drawing on the COR theory, the results showed that leader expressed humility did have a dark side, and the extent to which this dark side shows up rests with whether they are acting counterdispositionally. We hope that our work not only shifts the focus of expressed humility research toward the outcomes for the leaders themselves but also fuels scholars’ interest to further explore the pros and cons of leader expressed humility.

## Data Availability

The datasets generated for this study are available on request to the corresponding author.

## Ethics Statement

An ethics approval was not required as per institutional guidelines and national laws and regulations, because no unethical behaviors existed in this study. We just conducted a paper-pencil test and were exempt from further ethics board approval since our study did not involve human clinical trials or animal experiments. In the survey process, all participants were informed that participation was voluntary and assured that their responses would be used only for our research and kept strictly confidential. Therefore, only those who were willing to participate were recruited. The informed consent of the participants was implied through survey completion. To ensure confidentiality, the questionnaires completed during their working hours were directly returned to the research assistants in sealed envelopes.

## Author Contributions

ZL and ZW made substantial contributions to the conception and design of the studies. KY and LZ were responsible for collecting and analyzing the data. KY drafted the manuscript. All authors critically revised the manuscript for important intellectual content.

## Conflict of Interest Statement

The authors declare that the research was conducted in the absence of any commercial or financial relationships that could be construed as a potential conflict of interest.
